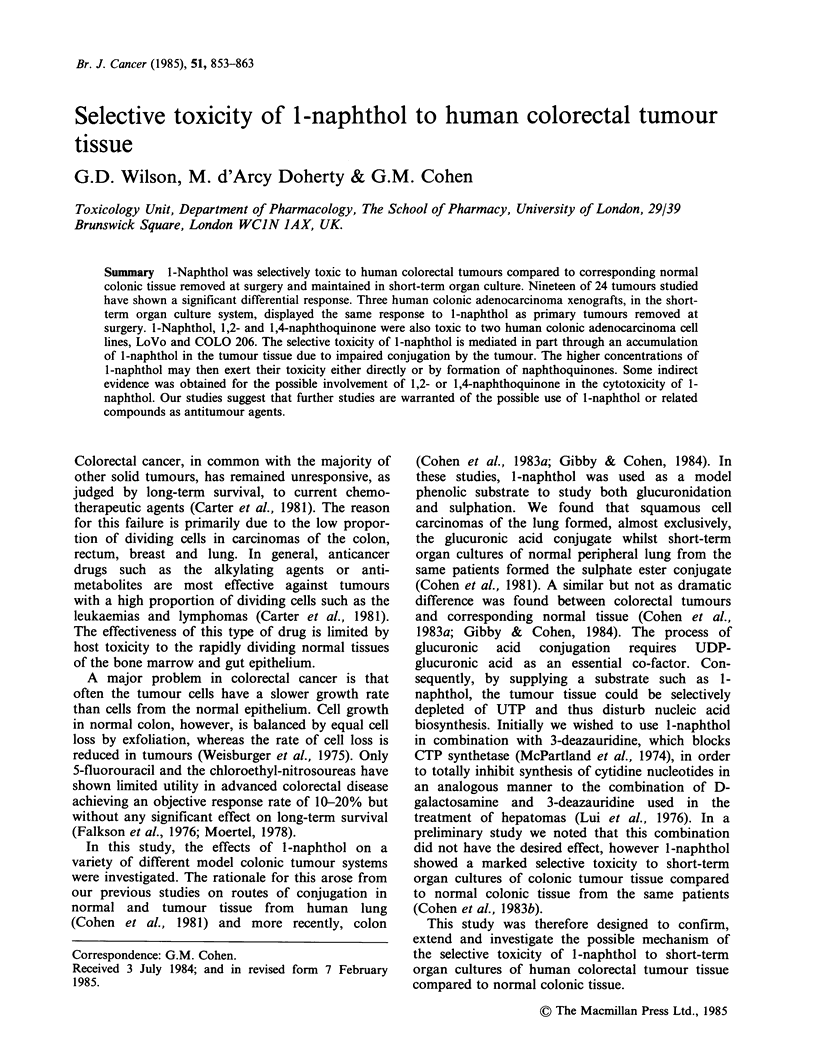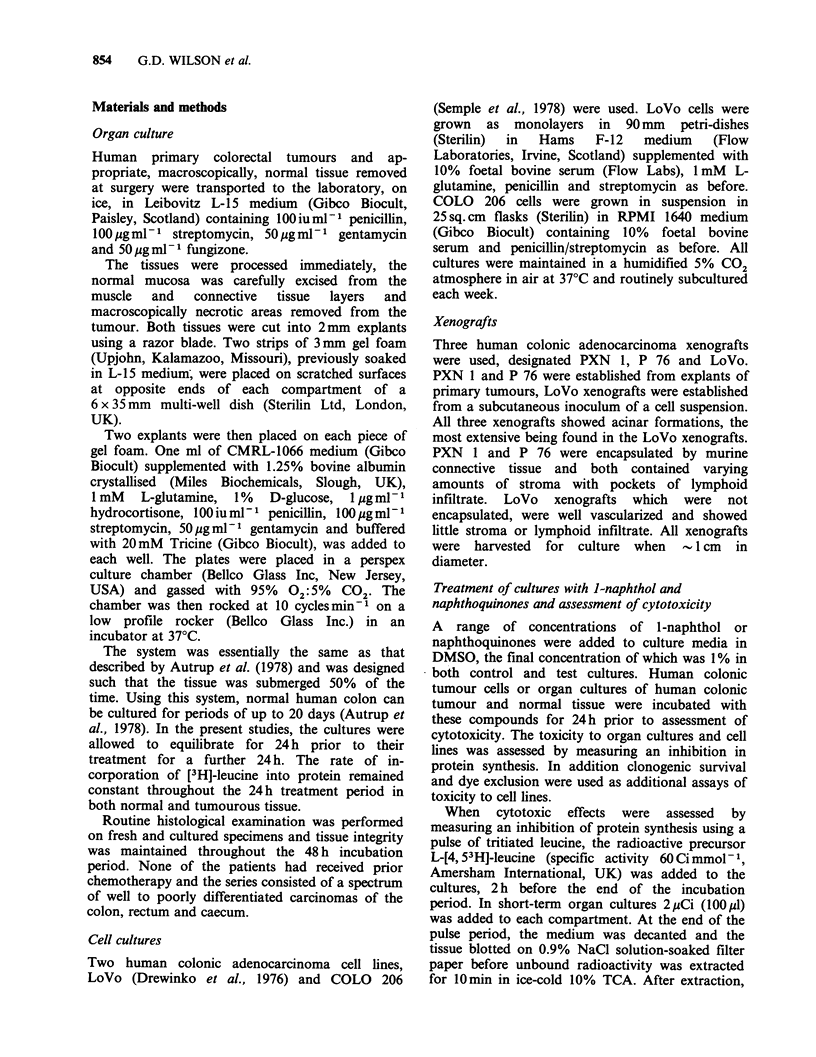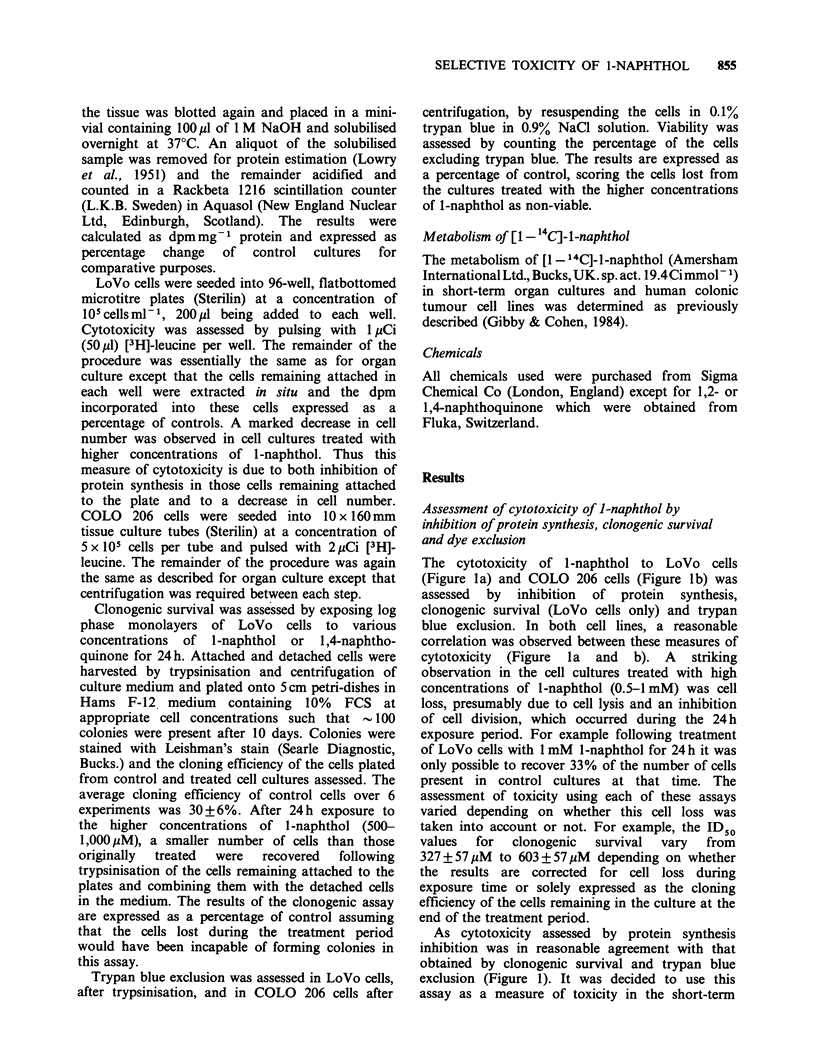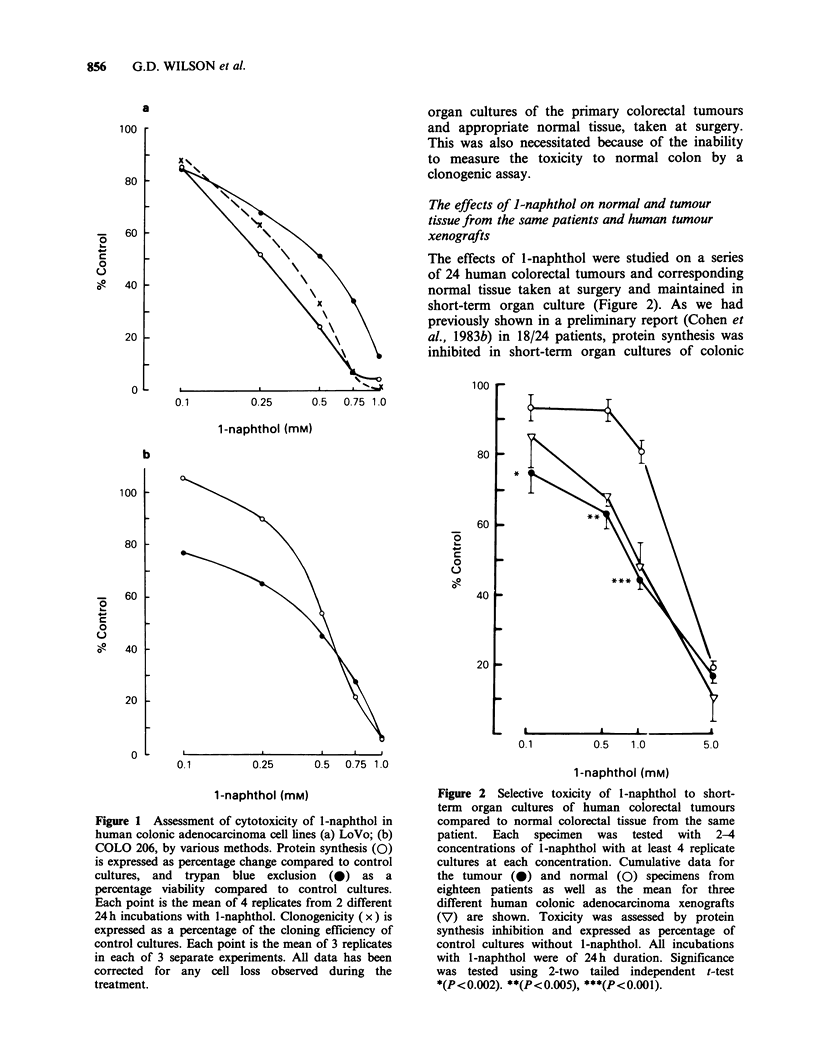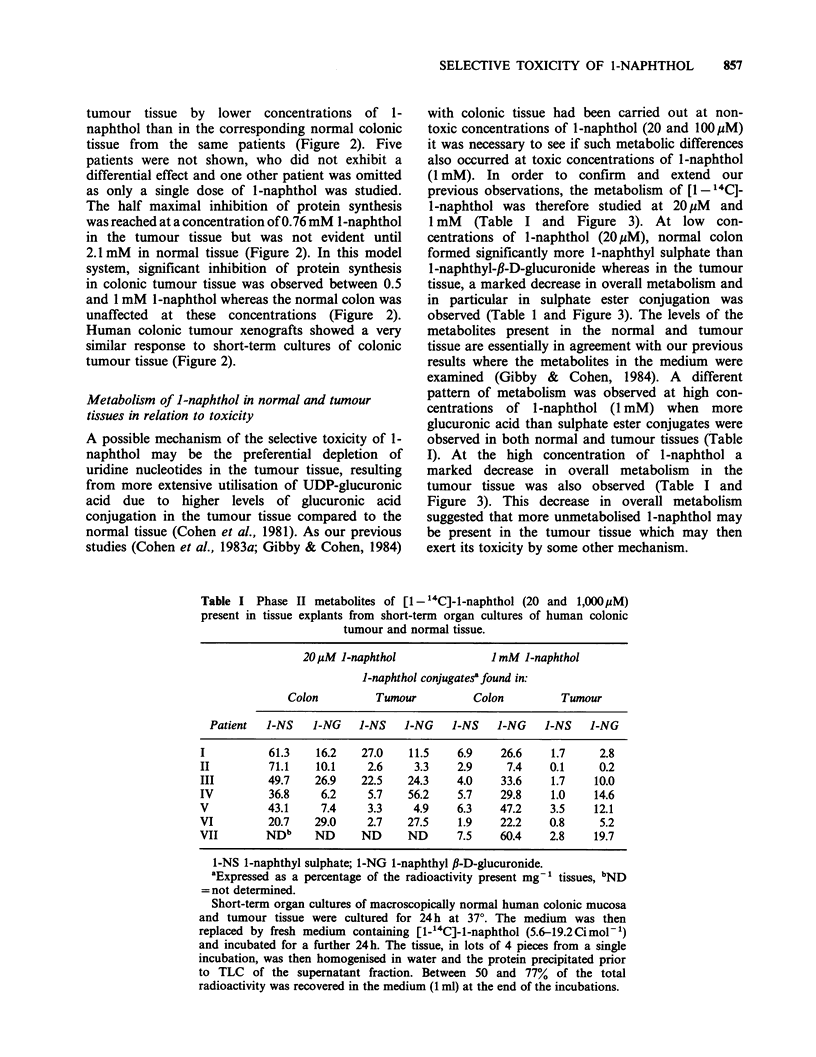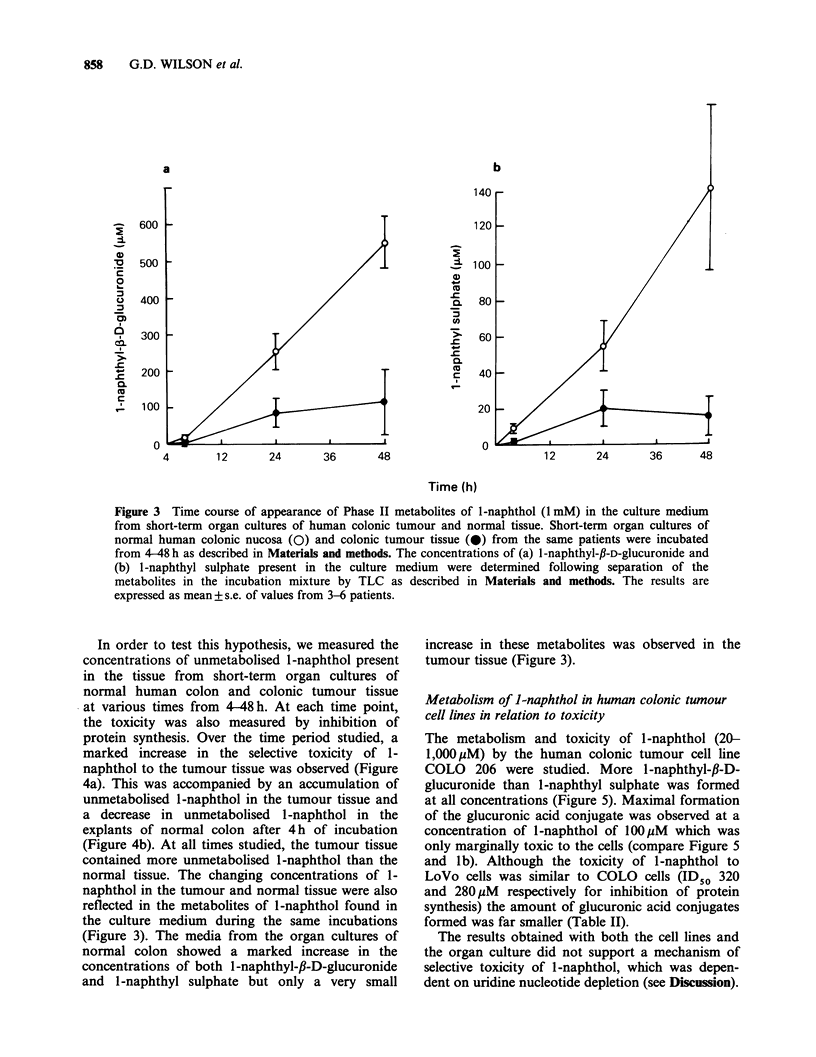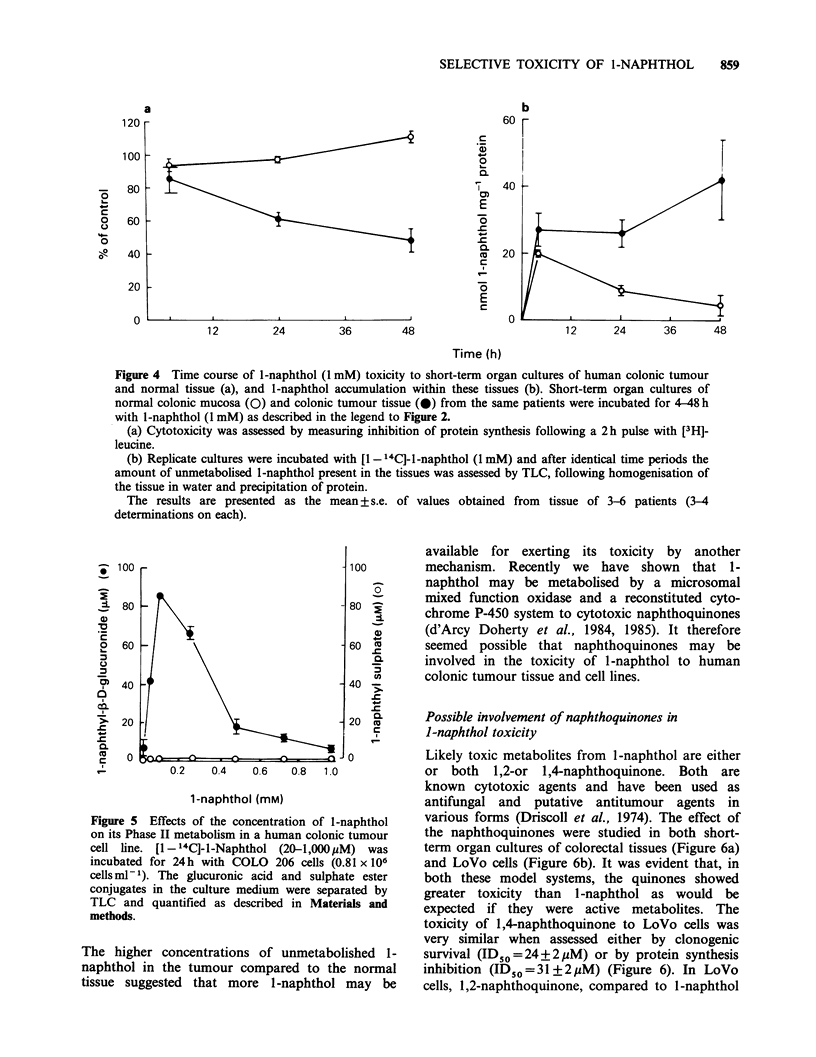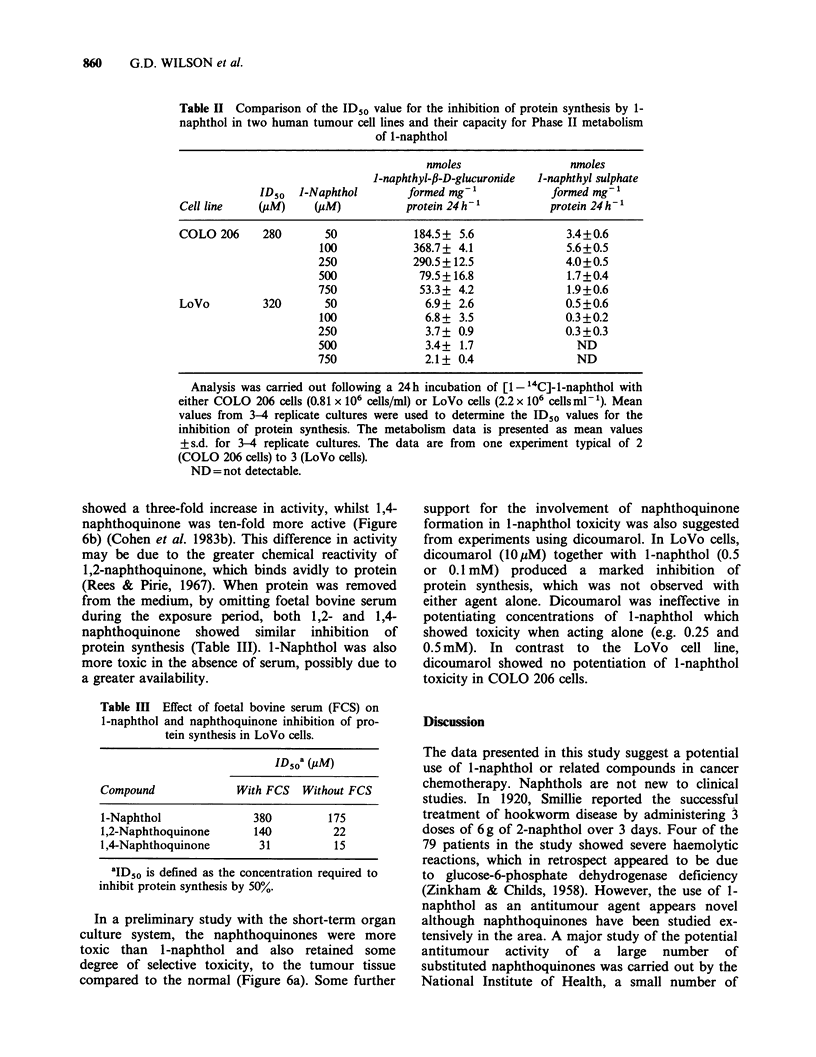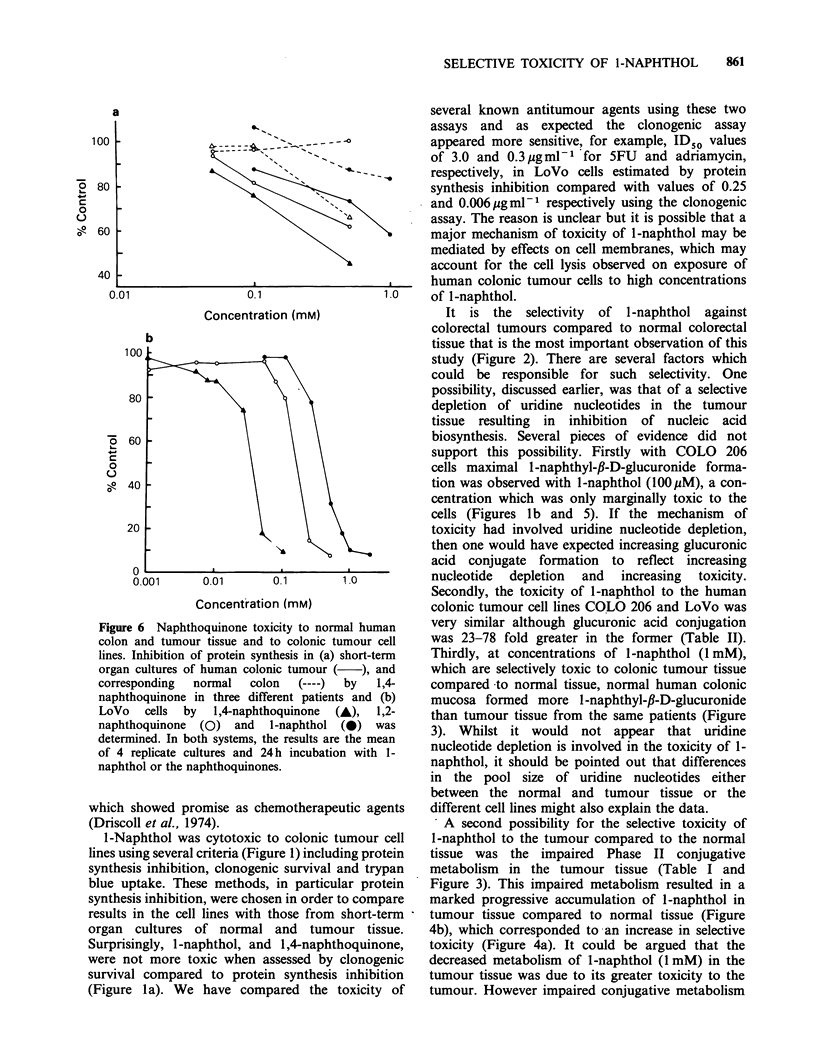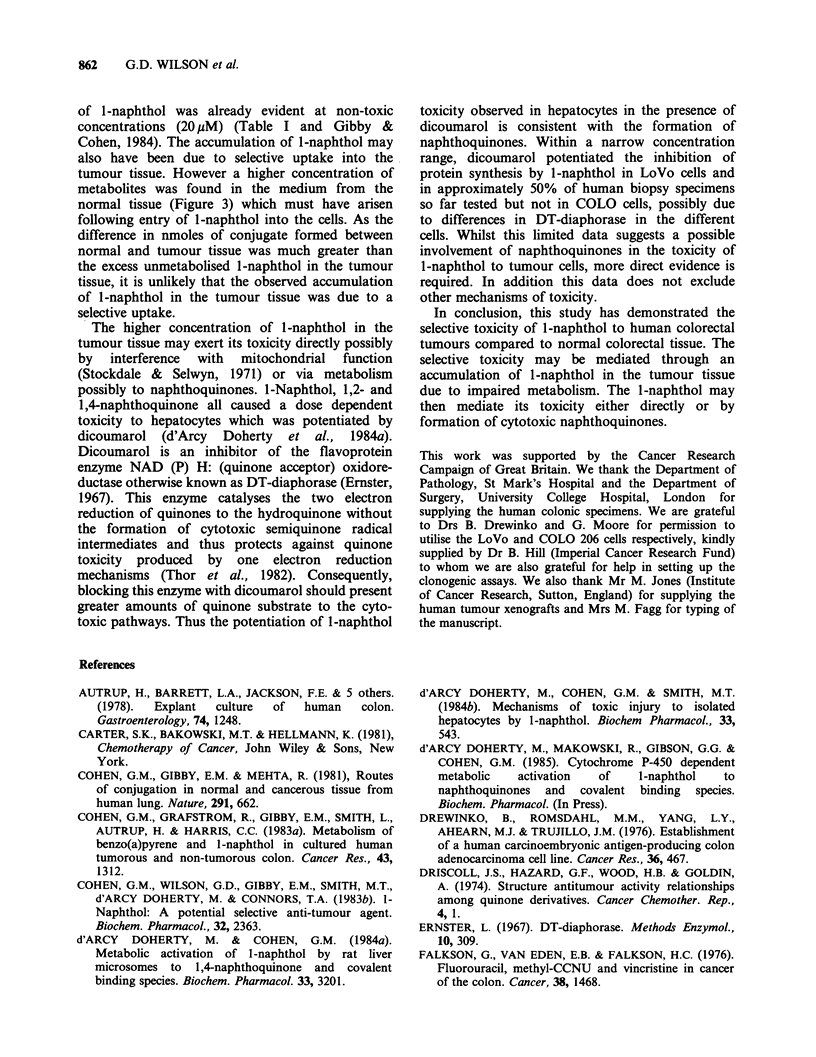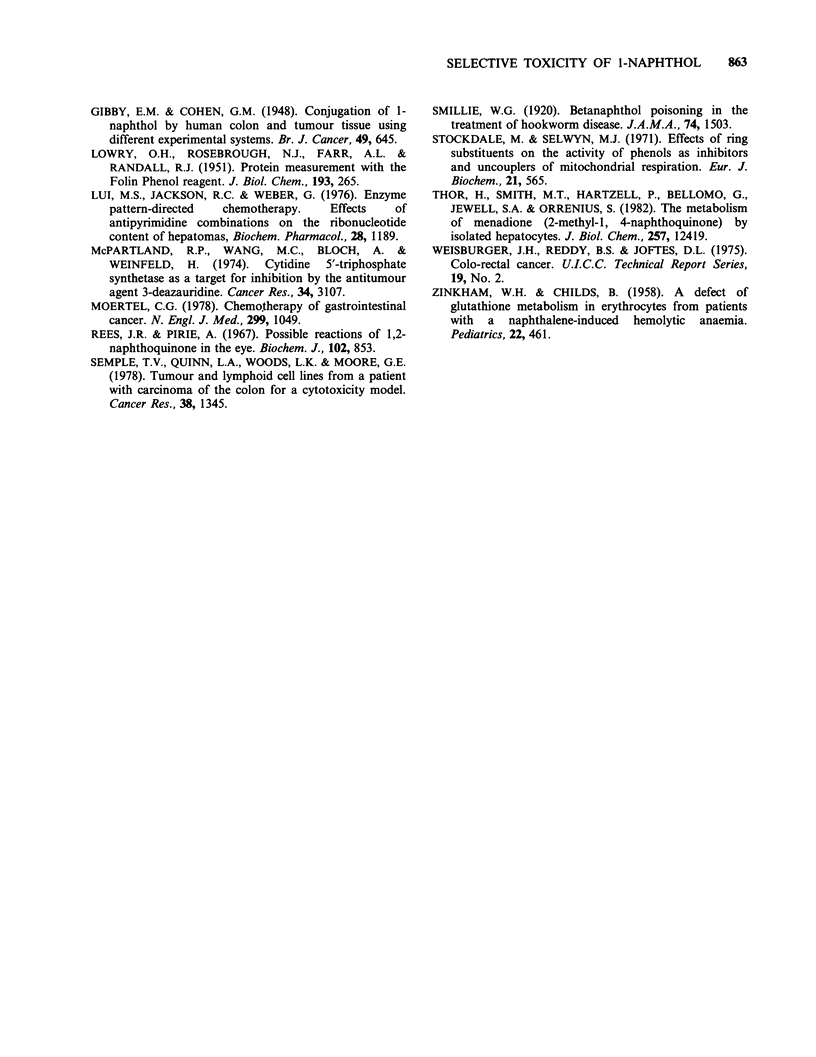# Selective toxicity of 1-naphthol to human colorectal tumour tissue.

**DOI:** 10.1038/bjc.1985.131

**Published:** 1985-06

**Authors:** G. D. Wilson, M. d'Arcy Doherty, G. M. Cohen

## Abstract

1-Naphthol was selectively toxic to human colorectal tumours compared to corresponding normal colonic tissue removed at surgery and maintained in short-term organ culture. Nineteen of 24 tumours studied have shown a significant differential response. Three human colonic adenocarcinoma xenografts, in the short-term organ culture system, displayed the same response to 1-naphthol as primary tumours removed at surgery. 1-Naphthol, 1,2- and 1,4-naphthoquinone were also toxic to two human colonic adenocarcinoma cell lines, LoVo and COLO 206. The selective toxicity of 1-naphthol is mediated in part through an accumulation of 1-naphthol in the tumour tissue due to impaired conjugation by the tumour. The higher concentrations of 1-naphthol may then exert their toxicity either directly or by formation of naphthoquinones. Some indirect evidence was obtained for the possible involvement of 1,2- or 1,4-naphthoquinone in the cytotoxicity of 1-naphthol. Our studies suggest that further studies are warranted of the possible use of 1-naphthol or related compounds as antitumour agents.


*Br. J. Cancer* (1985), **51**, 853–863

### Selective toxicity of 1-naphthol to human colorectal tumour tissue

G.D. Wilson, M. d'Arcy Doherty & G.M. Cohen

*Toxicology Unit, Department of Pharmacology, The School of Pharmacy, University of London, 29/39 Brunswick Square, London WC1N 1AX, UK.*

**Summary** 1-Naphthol was selectively toxic to human colorectal tumours compared to corresponding normal colonic tissue removed at surgery and maintained in short-term organ culture. Nineteen of 24 tumours studied have shown a significant differential response. Three human colonic adenocarcinoma xenografts, in the short-term organ culture system, displayed the same response to 1-naphthol as primary tumours removed at surgery. 1-Naphthol, 1,2- and 1,4-naphthoquinone were also toxic to two human colonic adenocarcinoma cell lines, LoVo and COLO 206. The selective toxicity of 1-naphthol is mediated in part through an accumulation of 1-naphthol in the tumour tissue due to impaired conjugation by the tumour. The higher concentrations of 1-naphthol may then exert their toxicity either directly or by formation of naphthoquinones. Some indirect evidence was obtained for the possible involvement of 1,2- or 1,4-naphthoquinone in the cytotoxicity of 1-naphthol. Our studies suggest that further studies are warranted of the possible use of 1-naphthol or related compounds as antitumour agents.

Colorectal cancer, in common with the majority of other solid tumours, has remained unresponsive, as judged by long-term survival, to current chemotherapeutic agents (Carter *et al.*, 1981). The reason for this failure is primarily due to the low proportion of dividing cells in carcinomas of the colon, rectum, breast and lung. In general, anticancer drugs such as the alkylating agents or antimetabolites are most effective against tumours with a high proportion of dividing cells such as the leukaemias and lymphomas (Carter *et al.*, 1981). The effectiveness of this type of drug is limited by host toxicity to the rapidly dividing normal tissues of the bone marrow and gut epithelium.

A major problem in colorectal cancer is that often the tumour cells have a slower growth rate than cells from the normal epithelium. Cell growth in normal colon, however, is balanced by equal cell loss by exfoliation, whereas the rate of cell loss is reduced in tumours (Weisburger *et al.*, 1975). Only 5-fluorouracil and the chloroethyl-nitrosoureas have shown limited utility in advanced colorectal disease achieving an objective response rate of 10–20% but without any significant effect on long-term survival (Falkson *et al.*, 1976; Moertel, 1978).

In this study, the effects of 1-naphthol on a variety of different model colonic tumour systems were investigated. The rationale for this arose from our previous studies on routes of conjugation in normal and tumour tissue from human lung (Cohen *et al.*, 1981) and more recently, colon (Cohen *et al.*, 1983*a*; Gibby & Cohen, 1984). In these studies, 1-naphthol was used as a model phenolic substrate to study both glucuronidation and sulphation. We found that squamous cell carcinomas of the lung formed, almost exclusively, the glucuronic acid conjugate whilst short-term organ cultures of normal peripheral lung from the same patients formed the sulphate ester conjugate (Cohen *et al.*, 1981). A similar but not as dramatic difference was found between colorectal tumours and corresponding normal tissue (Cohen *et al.*, 1983*a*; Gibby & Cohen, 1984). The process of glucuronic acid conjugation requires UDP-glucuronic acid as an essential co-factor. Consequently, by supplying a substrate such as 1-naphthol, the tumour tissue could be selectively depleted of UTP and thus disturb nucleic acid biosynthesis. Initially we wished to use 1-naphthol in combination with 3-deazauridine, which blocks CTP synthetase (McPartland *et al.*, 1974), in order to totally inhibit synthesis of cytidine nucleotides in an analogous manner to the combination of D-galactosamine and 3-deazauridine used in the treatment of hepatomas (Lui *et al.*, 1976). In a preliminary study we noted that this combination did not have the desired effect, however 1-naphthol showed a marked selective toxicity to short-term organ cultures of colonic tumour tissue compared to normal colonic tissue from the same patients (Cohen *et al.*, 1983*b*).

This study was therefore designed to confirm, extend and investigate the possible mechanism of the selective toxicity of 1-naphthol to short-term organ cultures of human colorectal tumour tissue compared to normal colonic tissue.

Correspondence: G.M. Cohen.
Received 3 July 1984; and in revised form 7 February 1985.

© The Macmillan Press Ltd., 1985

### Materials and methods

### Organ culture

Human primary colorectal tumours and appropriate, macroscopically, normal tissue removed at surgery were transported to the laboratory, on ice, in Leibovitz L-15 medium (Gibco Biocult, Paisley, Scotland) containing 100 iu ml$^{-1}$ penicillin, 100 $\mu$g ml$^{-1}$ streptomycin, 50 $\mu$g ml$^{-1}$ gentamycin and 50 $\mu$g ml$^{-1}$ fungizone.

The tissues were processed immediately, the normal mucosa was carefully excised from the muscle and connective tissue layers and macroscopically necrotic areas removed from the tumour. Both tissues were cut into 2 mm explants using a razor blade. Two strips of 3 mm gel foam (Upjohn, Kalamazoo, Missouri), previously soaked in L-15 medium, were placed on scratched surfaces at opposite ends of each compartment of a 6 × 35 mm multi-well dish (Sterilin Ltd, London, UK).

Two explants were then placed on each piece of gel foam. One ml of CMRL-1066 medium (Gibco Biocult) supplemented with 1.25% bovine albumin crystallised (Miles Biochemicals, Slough, UK), 1 mM L-glutamine, 1% D-glucose, 1 $\mu$g ml$^{-1}$ hydrocortisone, 100 iu ml$^{-1}$ penicillin, 100 $\mu$g ml$^{-1}$ streptomycin, 50 $\mu$g ml$^{-1}$ gentamycin and buffered with 20 mM Tricine (Gibco Biocult), was added to each well. The plates were placed in a perspex culture chamber (Bellco Glass Inc, New Jersey, USA) and gassed with 95% $O_2$:5% $CO_2$. The chamber was then rocked at 10 cycles min$^{-1}$ on a low profile rocker (Bellco Glass Inc.) in an incubator at 37°C.

The system was essentially the same as that described by Autrup *et al.* (1978) and was designed such that the tissue was submerged 50% of the time. Using this system, normal human colon can be cultured for periods of up to 20 days (Autrup *et al.*, 1978). In the present studies, the cultures were allowed to equilibrate for 24 h prior to their treatment for a further 24 h. The rate of incorporation of [$^3$H]-leucine into protein remained constant throughout the 24 h treatment period in both normal and tumourous tissue.

Routine histological examination was performed on fresh and cultured specimens and tissue integrity was maintained throughout the 48 h incubation period. None of the patients had received prior chemotherapy and the series consisted of a spectrum of well to poorly differentiated carcinomas of the colon, rectum and caecum.

### Cell cultures

Two human colonic adenocarcinoma cell lines, LoVo (Drewinko *et al.*, 1976) and COLO 206 (Semple *et al.*, 1978) were used. LoVo cells were grown as monolayers in 90 mm petri-dishes (Sterilin) in Hams F-12 medium (Flow Laboratories, Irvine, Scotland) supplemented with 10% foetal bovine serum (Flow Labs), 1 mM L-glutamine, penicillin and streptomycin as before. COLO 206 cells were grown in suspension in 25 sq. cm flasks (Sterilin) in RPMI 1640 medium (Gibco Biocult) containing 10% foetal bovine serum and penicillin/streptomycin as before. All cultures were maintained in a humidified 5% $CO_2$ atmosphere in air at 37°C and routinely subcultured each week.

### Xenografts

Three human colonic adenocarcinoma xenografts were used, designated PXN 1, P 76 and LoVo. PXN 1 and P 76 were established from explants of primary tumours, LoVo xenografts were established from a subcutaneous inoculum of a cell suspension. All three xenografts showed acinar formations, the most extensive being found in the LoVo xenografts. PXN 1 and P 76 were encapsulated by murine connective tissue and both contained varying amounts of stroma with pockets of lymphoid infiltrate. LoVo xenografts which were not encapsulated, were well vascularized and showed little stroma or lymphoid infiltrate. All xenografts were harvested for culture when ~1 cm in diameter.

### Treatment of cultures with 1-naphthol and naphthoquinones and assessment of cytotoxicity

A range of concentrations of 1-naphthol or naphthoquinones were added to culture media in DMSO, the final concentration of which was 1% in both control and test cultures. Human colonic tumour cells or organ cultures of human colonic tumour and normal tissue were incubated with these compounds for 24 h prior to assessment of cytotoxicity. The toxicity to organ cultures and cell lines was assessed by measuring an inhibition in protein synthesis. In addition clonogenic survival and dye exclusion were used as additional assays of toxicity to cell lines.

When cytotoxic effects were assessed by measuring an inhibition of protein synthesis using a pulse of tritiated leucine, the radioactive precursor L-[4, 5$^3$H]-leucine (specific activity 60 Ci mmol$^{-1}$, Amersham International, UK) was added to the cultures, 2 h before the end of the incubation period. In short-term organ cultures 2 $\mu$Ci (100 $\mu$l) was added to each compartment. At the end of the pulse period, the medium was decanted and the tissue blotted on 0.9% NaCl solution-soaked filter paper before unbound radioactivity was extracted for 10 min in ice-cold 10% TCA. After extraction, the tissue was blotted again and placed in a mini-vial containing 100 $\mu$l of 1 M NaOH and solubilised overnight at 37°C. An aliquot of the solubilised sample was removed for protein estimation (Lowry *et al.,* 1951) and the remainder acidified and counted in a Rackbeta 1216 scintillation counter (L.K.B. Sweden) in Aquasol (New England Nuclear Ltd, Edinburgh, Scotland). The results were calculated as dpm mg$^{-1}$ protein and expressed as percentage change of control cultures for comparative purposes.

LoVo cells were seeded into 96-well, flatbottomed microtitre plates (Sterilin) at a concentration of $10^5$ cells ml$^{-1}$, 200 $\mu$l being added to each well. Cytotoxicity was assessed by pulsing with 1 $\mu$Ci (50 $\mu$l) [$^3$H]-leucine per well. The remainder of the procedure was essentially the same as for organ culture except that the cells remaining attached in each well were extracted *in situ* and the dpm incorporated into these cells expressed as a percentage of controls. A marked decrease in cell number was observed in cell cultures treated with higher concentrations of 1-naphthol. Thus this measure of cytotoxicity is due to both inhibition of protein synthesis in those cells remaining attached to the plate and to a decrease in cell number. COLO 206 cells were seeded into 10 x 160 mm tissue culture tubes (Sterilin) at a concentration of $5 \times 10^5$ cells per tube and pulsed with 2 $\mu$Ci [$^3$H]-leucine. The remainder of the procedure was again the same as described for organ culture except that centrifugation was required between each step.

Clonogenic survival was assessed by exposing log phase monolayers of LoVo cells to various concentrations of 1-naphthol or 1,4-naphthoquinone for 24 h. Attached and detached cells were harvested by trypsinisation and centrifugation of culture medium and plated onto 5 cm petri-dishes in Hams F-12 medium containing 10% FCS at appropriate cell concentrations such that ~100 colonies were present after 10 days. Colonies were stained with Leishman's stain (Searle Diagnostic, Bucks.) and the cloning efficiency of the cells plated from control and treated cell cultures assessed. The average cloning efficiency of control cells over 6 experiments was 30 ± 6%. After 24 h exposure to the higher concentrations of 1-naphthol (500–1,000 $\mu$M), a smaller number of cells than those originally treated were recovered following trypsinisation of the cells remaining attached to the plates and combining them with the detached cells in the medium. The results of the clonogenic assay are expressed as a percentage of control assuming that the cells lost during the treatment period would have been incapable of forming colonies in this assay.

Trypan blue exclusion was assessed in LoVo cells, after trypsinisation, and in COLO 206 cells after centrifugation, by resuspending the cells in 0.1% trypan blue in 0.9% NaCl solution. Viability was assessed by counting the percentage of the cells excluding trypan blue. The results are expressed as a percentage of control, scoring the cells lost from the cultures treated with the higher concentrations of 1-naphthol as non-viable.

### *Metabolism of* [1 – $^{14}$C]-1-*naphthol*

The metabolism of [1 – $^{14}$C]-1-naphthol (Amersham International Ltd., Bucks, UK. sp. act. 19.4 Ci mmol$^{-1}$) in short-term organ cultures and human colonic tumour cell lines was determined as previously described (Gibby & Cohen, 1984).

### *Chemicals*

All chemicals used were purchased from Sigma Chemical Co (London, England) except for 1,2- or 1,4-naphthoquinone which were obtained from Fluka, Switzerland.

### Results

### *Assessment of cytotoxicity of 1-naphthol by inhibition of protein synthesis, clonogenic survival and dye exclusion*

The cytotoxicity of 1-naphthol to LoVo cells (Figure 1a) and COLO 206 cells (Figure 1b) was assessed by inhibition of protein synthesis, clonogenic survival (LoVo cells only) and trypan blue exclusion. In both cell lines, a reasonable correlation was observed between these measures of cytotoxicity (Figure 1a and b). A striking observation in the cell cultures treated with high concentrations of 1-naphthol (0.5–1 mM) was cell loss, presumably due to cell lysis and an inhibition of cell division, which occurred during the 24 h exposure period. For example following treatment of LoVo cells with 1 mM 1-naphthol for 24 h it was only possible to recover 33% of the number of cells present in control cultures at that time. The assessment of toxicity using each of these assays varied depending on whether this cell loss was taken into account or not. For example, the $ID_{50}$ values for clonogenic survival vary from 327 ± 57 $\mu$M to 603 ± 57 $\mu$M depending on whether the results are corrected for cell loss during exposure time or solely expressed as the cloning efficiency of the cells remaining in the culture at the end of the treatment period.

As cytotoxicity assessed by protein synthesis inhibition was in reasonable agreement with that obtained by clonogenic survival and trypan blue exclusion (Figure 1). It was decided to use this assay as a measure of toxicity in the short-term

**Figure 1** Assessment of cytotoxicity of 1-naphthol in human colonic adenocarcinoma cell lines (a) LoVo; (b) COLO 206, by various methods. Protein synthesis (○) is expressed as percentage change compared to control cultures, and trypan blue exclusion (●) as a percentage viability compared to control cultures. Each point is the mean of 4 replicates from 2 different 24 h incubations with 1-naphthol. Clonogenicity (×) is expressed as a percentage of the cloning efficiency of control cultures. Each point is the mean of 3 replicates in each of 3 separate experiments. All data has been corrected for any cell loss observed during the treatment.

organ cultures of the primary colorectal tumours and appropriate normal tissue, taken at surgery. This was also necessitated because of the inability to measure the toxicity to normal colon by a clonogenic assay.

### *The effects of 1-naphthol on normal and tumour tissue from the same patients and human tumour xenografts*

The effects of 1-naphthol were studied on a series of 24 human colorectal tumours and corresponding normal tissue taken at surgery and maintained in short-term organ culture (Figure 2). As we had previously shown in a preliminary report (Cohen *et al.*, 1983*b*) in 18/24 patients, protein synthesis was inhibited in short-term organ cultures of colonic

**Figure 2** Selective toxicity of 1-naphthol to short-term organ cultures of human colorectal tumours compared to normal colorectal tissue from the same patient. Each specimen was tested with 2–4 concentrations of 1-naphthol with at least 4 replicate cultures at each concentration. Cumulative data for the tumour (●) and normal (○) specimens from eighteen patients as well as the mean for three different human colonic adenocarcinoma xenografts (▽) are shown. Toxicity was assessed by protein synthesis inhibition and expressed as percentage of control cultures without 1-naphthol. All incubations with 1-naphthol were of 24 h duration. Significance was tested using 2-two tailed independent *t*-test *($P<0.002$). **($P<0.005$), ***($P<0.001$).

tumour tissue by lower concentrations of 1-naphthol than in the corresponding normal colonic tissue from the same patients (Figure 2). Five patients were not shown, who did not exhibit a differential effect and one other patient was omitted as only a single dose of 1-naphthol was studied. The half maximal inhibition of protein synthesis was reached at a concentration of 0.76 mM 1-naphthol in the tumour tissue but was not evident until 2.1 mM in normal tissue (Figure 2). In this model system, significant inhibition of protein synthesis in colonic tumour tissue was observed between 0.5 and 1 mM 1-naphthol whereas the normal colon was unaffected at these concentrations (Figure 2). Human colonic tumour xenografts showed a very similar response to short-term cultures of colonic tumour tissue (Figure 2).

### *Metabolism of 1-naphthol in normal and tumour tissues in relation to toxicity*

A possible mechanism of the selective toxicity of 1-naphthol may be the preferential depletion of uridine nucleotides in the tumour tissue, resulting from more extensive utilisation of UDP-glucuronic acid due to higher levels of glucuronic acid conjugation in the tumour tissue compared to the normal tissue (Cohen *et al.*, 1981). As our previous studies (Cohen *et al.*, 1983*a*; Gibby & Cohen, 1984) with colonic tissue had been carried out at non-toxic concentrations of 1-naphthol (20 and 100 $\mu$M) it was necessary to see if such metabolic differences also occurred at toxic concentrations of 1-naphthol (1 mM). In order to confirm and extend our previous observations, the metabolism of [$1-{}^{14}C$]-1-naphthol was therefore studied at 20 $\mu$M and 1 mM (Table I and Figure 3). At low concentrations of 1-naphthol (20 $\mu$M), normal colon formed significantly more 1-naphthyl sulphate than 1-naphthyl-$\beta$-D-glucuronide whereas in the tumour tissue, a marked decrease in overall metabolism and in particular in sulphate ester conjugation was observed (Table 1 and Figure 3). The levels of the metabolites present in the normal and tumour tissue are essentially in agreement with our previous results where the metabolites in the medium were examined (Gibby & Cohen, 1984). A different pattern of metabolism was observed at high concentrations of 1-naphthol (1 mM) when more glucuronic acid than sulphate ester conjugates were observed in both normal and tumour tissues (Table I). At the high concentration of 1-naphthol a marked decrease in overall metabolism in the tumour tissue was also observed (Table I and Figure 3). This decrease in overall metabolism suggested that more unmetabolised 1-naphthol may be present in the tumour tissue which may then exert its toxicity by some other mechanism.

**Table I** Phase II metabolites of [$1-{}^{14}C$]-1-naphthol (20 and 1,000 $\mu$M) present in tissue explants from short-term organ cultures of human colonic tumour and normal tissue.

| | *20 µM 1-naphthol* | | | | *1 mM 1-naphthol* | | | |
|---|---|---|---|---|---|---|---|---|
| | *1-naphthol conjugates[a] found in:* | | | | | | | |
| | *Colon* | | *Tumour* | | *Colon* | | *Tumour* | |
| *Patient* | *1-NS* | *1-NG* | *1-NS* | *1-NG* | *1-NS* | *1-NG* | *1-NS* | *1-NG* |
| I | 61.3 | 16.2 | 27.0 | 11.5 | 6.9 | 26.6 | 1.7 | 2.8 |
| II | 71.1 | 10.1 | 2.6 | 3.3 | 2.9 | 7.4 | 0.1 | 0.2 |
| III | 49.7 | 26.9 | 22.5 | 24.3 | 4.0 | 33.6 | 1.7 | 10.0 |
| IV | 36.8 | 6.2 | 5.7 | 56.2 | 5.7 | 29.8 | 1.0 | 14.6 |
| V | 43.1 | 7.4 | 3.3 | 4.9 | 6.3 | 47.2 | 3.5 | 12.1 |
| VI | 20.7 | 29.0 | 2.7 | 27.5 | 1.9 | 22.2 | 0.8 | 5.2 |
| VII | ND[b] | ND | ND | ND | 7.5 | 60.4 | 2.8 | 19.7 |

1-NS 1-naphthyl sulphate; 1-NG 1-naphthyl $\beta$-D-glucuronide.

[a]Expressed as a percentage of the radioactivity present $mg^{-1}$ tissues, [b]ND = not determined.

Short-term organ cultures of macroscopically normal human colonic mucosa and tumour tissue were cultured for 24 h at 37°. The medium was then replaced by fresh medium containing [$1-{}^{14}C$]-1-naphthol (5.6–19.2 Ci $mol^{-1}$) and incubated for a further 24 h. The tissue, in lots of 4 pieces from a single incubation, was then homogenised in water and the protein precipitated prior to TLC of the supernatant fraction. Between 50 and 77% of the total radioactivity was recovered in the medium (1 ml) at the end of the incubations.

**Figure 3** Time course of appearance of Phase II metabolites of 1-naphthol (1 mM) in the culture medium from short-term organ cultures of human colonic tumour and normal tissue. Short-term organ cultures of normal human colonic nucosa (○) and colonic tumour tissue (●) from the same patients were incubated from 4–48 h as described in **Materials and methods.** The concentrations of (a) 1-naphthyl-β-D-glucuronide and (b) 1-naphthyl sulphate present in the culture medium were determined following separation of the metabolites in the incubation mixture by TLC as described in **Materials and methods.** The results are expressed as mean ± s.e. of values from 3–6 patients.

In order to test this hypothesis, we measured the concentrations of unmetabolised 1-naphthol present in the tissue from short-term organ cultures of normal human colon and colonic tumour tissue at various times from 4–48 h. At each time point, the toxicity was also measured by inhibition of protein synthesis. Over the time period studied, a marked increase in the selective toxicity of 1-naphthol to the tumour tissue was observed (Figure 4a). This was accompanied by an accumulation of unmetabolised 1-naphthol in the tumour tissue and a decrease in unmetabolised 1-naphthol in the explants of normal colon after 4 h of incubation (Figure 4b). At all times studied, the tumour tissue contained more unmetabolised 1-naphthol than the normal tissue. The changing concentrations of 1-naphthol in the tumour and normal tissue were also reflected in the metabolites of 1-naphthol found in the culture medium during the same incubations (Figure 3). The media from the organ cultures of normal colon showed a marked increase in the concentrations of both 1-naphthyl-β-D-glucuronide and 1-naphthyl sulphate but only a very small increase in these metabolites was observed in the tumour tissue (Figure 3).

*Metabolism of 1-naphthol in human colonic tumour cell lines in relation to toxicity*

The metabolism and toxicity of 1-naphthol (20–1,000 μM) by the human colonic tumour cell line COLO 206 were studied. More 1-naphthyl-β-D-glucuronide than 1-naphthyl sulphate was formed at all concentrations (Figure 5). Maximal formation of the glucuronic acid conjugate was observed at a concentration of 1-naphthol of 100 μM which was only marginally toxic to the cells (compare Figure 5 and 1b). Although the toxicity of 1-naphthol to LoVo cells was similar to COLO cells ($ID_{50}$ 320 and 280 μM respectively for inhibition of protein synthesis) the amount of glucuronic acid conjugates formed was far smaller (Table II).

The results obtained with both the cell lines and the organ culture did not support a mechanism of selective toxicity of 1-naphthol, which was dependent on uridine nucleotide depletion (see **Discussion**).

**Figure 4** Time course of 1-naphthol (1 mM) toxicity to short-term organ cultures of human colonic tumour and normal tissue (a), and 1-naphthol accumulation within these tissues (b). Short-term organ cultures of normal colonic mucosa (○) and colonic tumour tissue (●) from the same patients were incubated for 4–48 h with 1-naphthol (1 mM) as described in the legend to **Figure 2.**

(a) Cytotoxicity was assessed by measuring inhibition of protein synthesis following a 2 h pulse with [$^3$H]-leucine.

(b) Replicate cultures were incubated with [$1-{}^{14}$C]-1-naphthol (1 mM) and after identical time periods the amount of unmetabolised 1-naphthol present in the tissues was assessed by TLC, following homogenisation of the tissue in water and precipitation of protein.

The results are presented as the mean ± s.e. of values obtained from tissue of 3–6 patients (3–4 determinations on each).

**Figure 5** Effects of the concentration of 1-naphthol on its Phase II metabolism in a human colonic tumour cell line. [$1-{}^{14}$C]-1-Naphthol (20–1,000 μM) was incubated for 24 h with COLO 206 cells ($0.81 \times 10^6$ cells ml$^{-1}$). The glucuronic acid and sulphate ester conjugates in the culture medium were separated by TLC and quantified as described in **Materials and methods.**

The higher concentrations of unmetabolised 1-naphthol in the tumour compared to the normal tissue suggested that more 1-naphthol may be available for exerting its toxicity by another mechanism. Recently we have shown that 1-naphthol may be metabolised by a microsomal mixed function oxidase and a reconstituted cytochrome P-450 system to cytotoxic naphthoquinones (d'Arcy Doherty *et al.*, 1984, 1985). It therefore seemed possible that naphthoquinones may be involved in the toxicity of 1-naphthol to human colonic tumour tissue and cell lines.

### *Possible involvement of naphthoquinones in 1-naphthol toxicity*

Likely toxic metabolites from 1-naphthol are either or both 1,2-or 1,4-naphthoquinone. Both are known cytotoxic agents and have been used as antifungal and putative antitumour agents in various forms (Driscoll *et al.*, 1974). The effect of the naphthoquinones were studied in both short-term organ cultures of colorectal tissues (Figure 6a) and LoVo cells (Figure 6b). It was evident that, in both these model systems, the quinones showed greater toxicity than 1-naphthol as would be expected if they were active metabolites. The toxicity of 1,4-naphthoquinone to LoVo cells was very similar when assessed either by clonogenic survival ($ID_{50} = 24 \pm 2$ μM) or by protein synthesis inhibition ($ID_{50} = 31 \pm 2$ μM) (Figure 6). In LoVo cells, 1,2-naphthoquinone, compared to 1-naphthol

**Table II** Comparison of the $ID_{50}$ value for the inhibition of protein synthesis by 1-naphthol in two human tumour cell lines and their capacity for Phase II metabolism of 1-naphthol

| *Cell line* | *$ID_{50}$ (μM)* | *1-Naphthol (μM)* | *nmoles 1-naphthyl-β-D-glucuronide formed $mg^{-1}$ protein 24 $h^{-1}$* | *nmoles 1-naphthyl sulphate formed $mg^{-1}$ protein 24 $h^{-1}$* |
|---|---|---|---|---|
| COLO 206 | 280 | 50 | 184.5 ± 5.6 | 3.4 ± 0.6 |
| | | 100 | 368.7 ± 4.1 | 5.6 ± 0.5 |
| | | 250 | 290.5 ± 12.5 | 4.0 ± 0.5 |
| | | 500 | 79.5 ± 16.8 | 1.7 ± 0.4 |
| | | 750 | 53.3 ± 4.2 | 1.9 ± 0.6 |
| LoVo | 320 | 50 | 6.9 ± 2.6 | 0.5 ± 0.6 |
| | | 100 | 6.8 ± 3.5 | 0.3 ± 0.2 |
| | | 250 | 3.7 ± 0.9 | 0.3 ± 0.3 |
| | | 500 | 3.4 ± 1.7 | ND |
| | | 750 | 2.1 ± 0.4 | ND |

Analysis was carried out following a 24 h incubation of $[1-{}^{14}C]$-1-naphthol with either COLO 206 cells ($0.81 \times 10^6$ cells/ml) or LoVo cells ($2.2 \times 10^6$ cells $ml^{-1}$). Mean values from 3–4 replicate cultures were used to determine the $ID_{50}$ values for the inhibition of protein synthesis. The metabolism data is presented as mean values ± s.d. for 3–4 replicate cultures. The data are from one experiment typical of 2 (COLO 206 cells) to 3 (LoVo cells).
ND = not detectable.

showed a three-fold increase in activity, whilst 1,4-naphthoquinone was ten-fold more active (Figure 6b) (Cohen *et al.* 1983b). This difference in activity may be due to the greater chemical reactivity of 1,2-naphthoquinone, which binds avidly to protein (Rees & Pirie, 1967). When protein was removed from the medium, by omitting foetal bovine serum during the exposure period, both 1,2- and 1,4-naphthoquinone showed similar inhibition of protein synthesis (Table III). 1-Naphthol was also more toxic in the absence of serum, possibly due to a greater availability.

**Table III** Effect of foetal bovine serum (FCS) on 1-naphthol and naphthoquinone inhibition of protein synthesis in LoVo cells.

| *Compound* | *$ID_{50}$[a] (μM)* With FCS | *Without FCS* |
|---|---|---|
| 1-Naphthol | 380 | 175 |
| 1,2-Naphthoquinone | 140 | 22 |
| 1,4-Naphthoquinone | 31 | 15 |

[a]$ID_{50}$ is defined as the concentration required to inhibit protein synthesis by 50%.

In a preliminary study with the short-term organ culture system, the naphthoquinones were more toxic than 1-naphthol and also retained some degree of selective toxicity, to the tumour tissue compared to the normal (Figure 6a). Some further support for the involvement of naphthoquinone formation in 1-naphthol toxicity was also suggested from experiments using dicoumarol. In LoVo cells, dicoumarol (10 μM) together with 1-naphthol (0.5 or 0.1 mM) produced a marked inhibition of protein synthesis, which was not observed with either agent alone. Dicoumarol was ineffective in potentiating concentrations of 1-naphthol which showed toxicity when acting alone (e.g. 0.25 and 0.5 mM). In contrast to the LoVo cell line, dicoumarol showed no potentiation of 1-naphthol toxicity in COLO 206 cells.

### Discussion

The data presented in this study suggest a potential use of 1-naphthol or related compounds in cancer chemotherapy. Naphthols are not new to clinical studies. In 1920, Smillie reported the successful treatment of hookworm disease by administering 3 doses of 6 g of 2-naphthol over 3 days. Four of the 79 patients in the study showed severe haemolytic reactions, which in retrospect appeared to be due to glucose-6-phosphate dehydrogenase deficiency (Zinkham & Childs, 1958). However, the use of 1-naphthol as an antitumour agent appears novel although naphthoquinones have been studied extensively in the area. A major study of the potential antitumour activity of a large number of substituted naphthoquinones was carried out by the National Institute of Health, a small number of

**Figure 6** Naphthoquinone toxicity to normal human colon and tumour tissue and to colonic tumour cell lines. Inhibition of protein synthesis in (a) short-term organ cultures of human colonic tumour (——), and corresponding normal colon (----) by 1,4-naphthoquinone in three different patients and (b) LoVo cells by 1,4-naphthoquinone (▲), 1,2-naphthoquinone (○) and 1-naphthol (●) was determined. In both systems, the results are the mean of 4 replicate cultures and 24 h incubation with 1-naphthol or the naphthoquinones.

which showed promise as chemotherapeutic agents (Driscoll *et al.*, 1974).

1-Naphthol was cytotoxic to colonic tumour cell lines using several criteria (Figure 1) including protein synthesis inhibition, clonogenic survival and trypan blue uptake. These methods, in particular protein synthesis inhibition, were chosen in order to compare results in the cell lines with those from short-term organ cultures of normal and tumour tissue. Surprisingly, 1-naphthol, and 1,4-naphthoquinone, were not more toxic when assessed by clonogenic survival compared to protein synthesis inhibition (Figure 1a). We have compared the toxicity of several known antitumour agents using these two assays and as expected the clonogenic assay appeared more sensitive, for example, $ID_{50}$ values of 3.0 and 0.3 $\mu g\, ml^{-1}$ for 5FU and adriamycin, respectively, in LoVo cells estimated by protein synthesis inhibition compared with values of 0.25 and 0.006 $\mu g\, ml^{-1}$ respectively using the clonogenic assay. The reason is unclear but it is possible that a major mechanism of toxicity of 1-naphthol may be mediated by effects on cell membranes, which may account for the cell lysis observed on exposure of human colonic tumour cells to high concentrations of 1-naphthol.

It is the selectivity of 1-naphthol against colorectal tumours compared to normal colorectal tissue that is the most important observation of this study (Figure 2). There are several factors which could be responsible for such selectivity. One possibility, discussed earlier, was that of a selective depletion of uridine nucleotides in the tumour tissue resulting in inhibition of nucleic acid biosynthesis. Several pieces of evidence did not support this possibility. Firstly with COLO 206 cells maximal 1-naphthyl-β-D-glucuronide formation was observed with 1-naphthol (100 μM), a concentration which was only marginally toxic to the cells (Figures 1b and 5). If the mechanism of toxicity had involved uridine nucleotide depletion, then one would have expected increasing glucuronic acid conjugate formation to reflect increasing nucleotide depletion and increasing toxicity. Secondly, the toxicity of 1-naphthol to the human colonic tumour cell lines COLO 206 and LoVo was very similar although glucuronic acid conjugation was 23–78 fold greater in the former (Table II). Thirdly, at concentrations of 1-naphthol (1 mM), which are selectively toxic to colonic tumour tissue compared to normal tissue, normal human colonic mucosa formed more 1-naphthyl-β-D-glucuronide than tumour tissue from the same patients (Figure 3). Whilst it would not appear that uridine nucleotide depletion is involved in the toxicity of 1-naphthol, it should be pointed out that differences in the pool size of uridine nucleotides either between the normal and tumour tissue or the different cell lines might also explain the data.

A second possibility for the selective toxicity of 1-naphthol to the tumour compared to the normal tissue was the impaired Phase II conjugative metabolism in the tumour tissue (Table I and Figure 3). This impaired metabolism resulted in a marked progressive accumulation of 1-naphthol in tumour tissue compared to normal tissue (Figure 4b), which corresponded to an increase in selective toxicity (Figure 4a). It could be argued that the decreased metabolism of 1-naphthol (1 mM) in the tumour tissue was due to its greater toxicity to the tumour. However impaired conjugative metabolism of 1-naphthol was already evident at non-toxic concentrations (20 $\mu$M) (Table I and Gibby & Cohen, 1984). The accumulation of 1-naphthol may also have been due to selective uptake into the tumour tissue. However a higher concentration of metabolites was found in the medium from the normal tissue (Figure 3) which must have arisen following entry of 1-naphthol into the cells. As the difference in nmoles of conjugate formed between normal and tumour tissue was much greater than the excess unmetabolised 1-naphthol in the tumour tissue, it is unlikely that the observed accumulation of 1-naphthol in the tumour tissue was due to a selective uptake.

The higher concentration of 1-naphthol in the tumour tissue may exert its toxicity directly possibly by interference with mitochondrial function (Stockdale & Selwyn, 1971) or via metabolism possibly to naphthoquinones. 1-Naphthol, 1,2- and 1,4-naphthoquinone all caused a dose dependent toxicity to hepatocytes which was potentiated by dicoumarol (d'Arcy Doherty *et al.*, 1984*a*). Dicoumarol is an inhibitor of the flavoprotein enzyme NAD (P) H: (quinone acceptor) oxidoreductase otherwise known as DT-diaphorase (Ernster, 1967). This enzyme catalyses the two electron reduction of quinones to the hydroquinone without the formation of cytotoxic semiquinone radical intermediates and thus protects against quinone toxicity produced by one electron reduction mechanisms (Thor *et al.*, 1982). Consequently, blocking this enzyme with dicoumarol should present greater amounts of quinone substrate to the cytotoxic pathways. Thus the potentiation of 1-naphthol toxicity observed in hepatocytes in the presence of dicoumarol is consistent with the formation of naphthoquinones. Within a narrow concentration range, dicoumarol potentiated the inhibition of protein synthesis by 1-naphthol in LoVo cells and in approximately 50% of human biopsy specimens so far tested but not in COLO cells, possibly due to differences in DT-diaphorase in the different cells. Whilst this limited data suggests a possible involvement of naphthoquinones in the toxicity of 1-naphthol to tumour cells, more direct evidence is required. In addition this data does not exclude other mechanisms of toxicity.

In conclusion, this study has demonstrated the selective toxicity of 1-naphthol to human colorectal tumours compared to normal colorectal tissue. The selective toxicity may be mediated through an accumulation of 1-naphthol in the tumour tissue due to impaired metabolism. The 1-naphthol may then mediate its toxicity either directly or by formation of cytotoxic naphthoquinones.

This work was supported by the Cancer Research Campaign of Great Britain. We thank the Department of Pathology, St Mark's Hospital and the Department of Surgery, University College Hospital, London for supplying the human colonic specimens. We are grateful to Drs B. Drewinko and G. Moore for permission to utilise the LoVo and COLO 206 cells respectively, kindly supplied by Dr B. Hill (Imperial Cancer Research Fund) to whom we are also grateful for help in setting up the clonogenic assays. We also thank Mr M. Jones (Institute of Cancer Research, Sutton, England) for supplying the human tumour xenografts and Mrs M. Fagg for typing of the manuscript.

### References

AUTRUP, H., BARRETT, L.A., JACKSON, F.E. & 5 others. (1978). Explant culture of human colon. *Gastroenterology*, **74**, 1248.

CARTER, S.K., BAKOWSKI, M.T. & HELLMANN, K. (1981), *Chemotherapy of Cancer*, John Wiley & Sons, New York.

COHEN, G.M., GIBBY, E.M. & MEHTA, R. (1981), Routes of conjugation in normal and cancerous tissue from human lung. *Nature*, **291**, 662.

COHEN, G.M., GRAFSTROM, R., GIBBY, E.M., SMITH, L., AUTRUP, H. & HARRIS, C.C. (1983*a*). Metabolism of benzo(a)pyrene and 1-naphthol in cultured human tumorous and non-tumorous colon. *Cancer Res.*, **43**, 1312.

COHEN, G.M., WILSON, G.D., GIBBY, E.M., SMITH, M.T., d'ARCY DOHERTY, M. & CONNORS, T.A. (1983*b*). 1-Naphthol: A potential selective anti-tumour agent. *Biochem. Pharmacol.*, **32**, 2363.

d'ARCY DOHERTY, M. & COHEN, G.M. (1984*a*). Metabolic activation of 1-naphthol by rat liver microsomes to 1,4-naphthoquinone and covalent binding species. *Biochem. Pharmacol.* **33**, 3201.

d'ARCY DOHERTY, M., COHEN, G.M. & SMITH, M.T. (1984*b*). Mechanisms of toxic injury to isolated hepatocytes by 1-naphthol. *Biochem Pharmacol.*, **33**, 543.

d'ARCY DOHERTY, M., MAKOWSKI, R., GIBSON, G.G. & COHEN, G.M. (1985). Cytochrome P-450 dependent metabolic activation of 1-naphthol to naphthoquinones and covalent binding species. *Biochem. Pharmacol.* (In Press).

DREWINKO, B., ROMSDAHL, M.M., YANG, L.Y., AHEARN, M.J. & TRUJILLO, J.M. (1976). Establishment of a human carcinoembryonic antigen-producing colon adenocarcinoma cell line. *Cancer Res.*, **36**, 467.

DRISCOLL, J.S., HAZARD, G.F., WOOD, H.B. & GOLDIN, A. (1974). Structure antitumour activity relationships among quinone derivatives. *Cancer Chemother. Rep.*, **4**, 1.

ERNSTER, L. (1967). DT-diaphorase. *Methods Enzymol.*, **10**, 309.

FALKSON, G., VAN EDEN, E.B. & FALKSON, H.C. (1976). Fluorouracil, methyl-CCNU and vincristine in cancer of the colon. *Cancer*, **38**, 1468.

GIBBY, E.M. & COHEN, G.M. (1948). Conjugation of 1-naphthol by human colon and tumour tissue using different experimental systems. *Br. J. Cancer*, **49**, 645.

LOWRY, O.H., ROSEBROUGH, N.J., FARR, A.L. & RANDALL, R.J. (1951). Protein measurement with the Folin Phenol reagent. *J. Biol. Chem.*, **193**, 265.

LUI, M.S., JACKSON, R.C. & WEBER, G. (1976). Enzyme pattern-directed chemotherapy. Effects of antipyrimidine combinations on the ribonucleotide content of hepatomas, *Biochem. Pharmacol.*, **28**, 1189.

McPARTLAND, R.P., WANG, M.C., BLOCH, A. & WEINFELD, H. (1974). Cytidine 5′-triphosphate synthetase as a target for inhibition by the antitumour agent 3-deazauridine. *Cancer Res.*, **34**, 3107.

MOERTEL, C.G. (1978). Chemotherapy of gastrointestinal cancer. *N. Engl. J. Med.*, **299**, 1049.

REES, J.R. & PIRIE, A. (1967). Possible reactions of 1,2-naphthoquinone in the eye. *Biochem. J.*, **102**, 853.

SEMPLE, T.V., QUINN, L.A., WOODS, L.K. & MOORE, G.E. (1978). Tumour and lymphoid cell lines from a patient with carcinoma of the colon for a cytotoxicity model. *Cancer Res.*, **38**, 1345.

SMILLIE, W.G. (1920). Betanaphthol poisoning in the treatment of hookworm disease. *J.A.M.A.*, **74**, 1503.

STOCKDALE, M. & SELWYN, M.J. (1971). Effects of ring substituents on the activity of phenols as inhibitors and uncouplers of mitochondrial respiration. *Eur. J. Biochem.*, **21**, 565.

THOR, H., SMITH, M.T., HARTZELL, P., BELLOMO, G., JEWELL, S.A. & ORRENIUS, S. (1982). The metabolism of menadione (2-methyl-1, 4-naphthoquinone) by isolated hepatocytes. *J. Biol. Chem.*, **257**, 12419.

WEISBURGER, J.H., REDDY, B.S. & JOFTES, D.L. (1975). Colo-rectal cancer. *U.I.C.C. Technical Report Series*, **19**, No. 2.

ZINKHAM, W.H. & CHILDS, B. (1958). A defect of glutathione metabolism in erythrocytes from patients with a naphthalene-induced hemolytic anaemia. *Pediatrics*, **22**, 461.